# Supplementary material for: RT-DOb, a switch gene for the gene pair {Csf1r, Milr1}, can influence the onset of Alzheimer’s disease by regulating communication between mast cell and microglia
Source: PLoS One. 2023 Jul 6;18(7):e0288134. doi: 10.1371/journal.pone.0288134 (PMC10325119; doi:10.1371/journal.pone.0288134)
Supplement: S1 Appendix — (PDF) [file pone.0288134.s001.pdf]

Supplementary Files:

S1 File. The presence of 18s- and 28s- rRNA bands on the electrophoresis gel confirmed the integrity of extracted RNA.

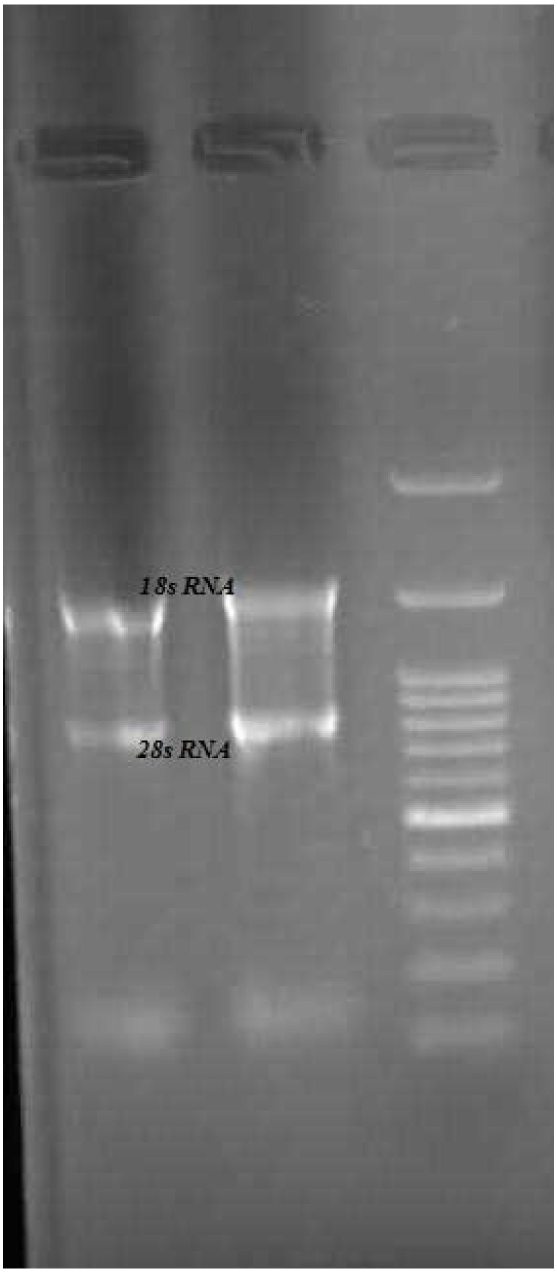

S2 File- Section A. Results of melting curve analysis.

Melting curve corresponding *Csf1r* gene.

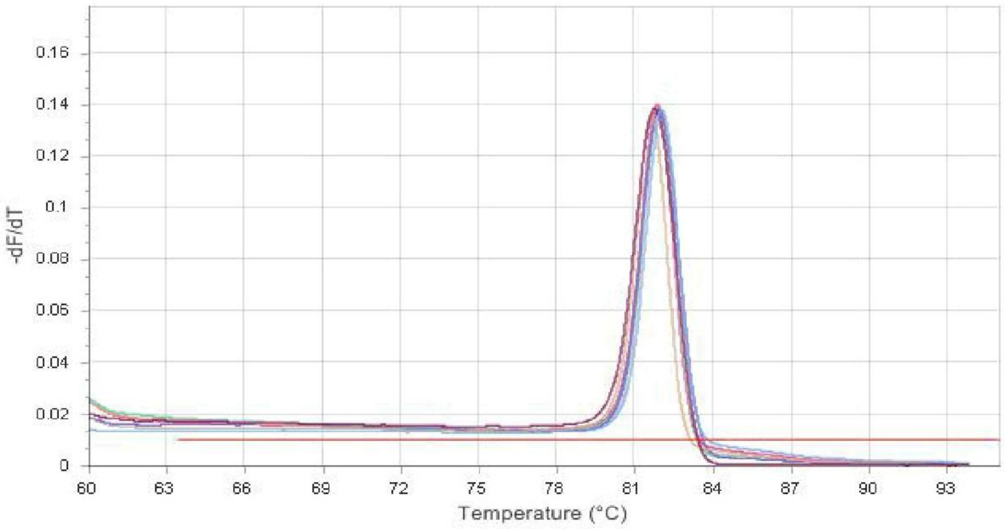

Melting curve corresponding *Milr1* gene.

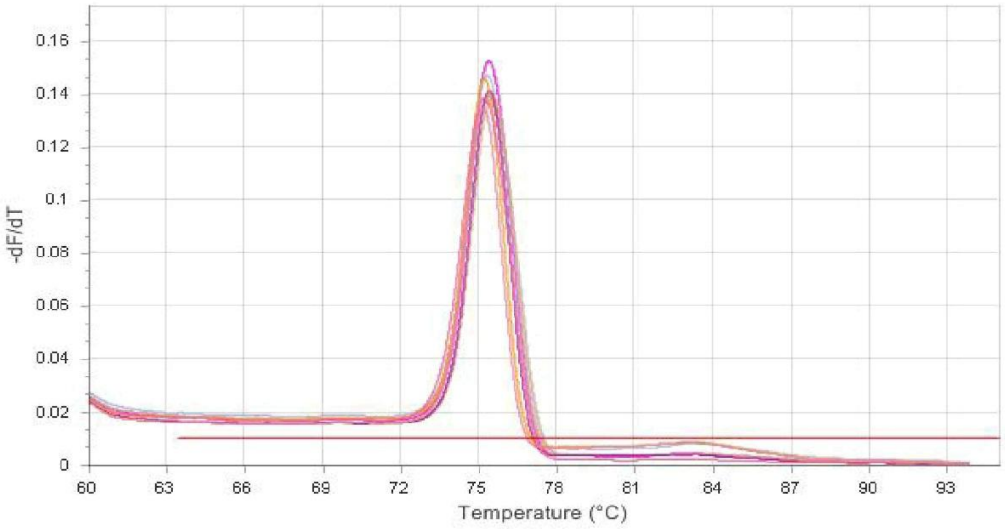

Melting curve corresponding *RT1-DOb* gene.

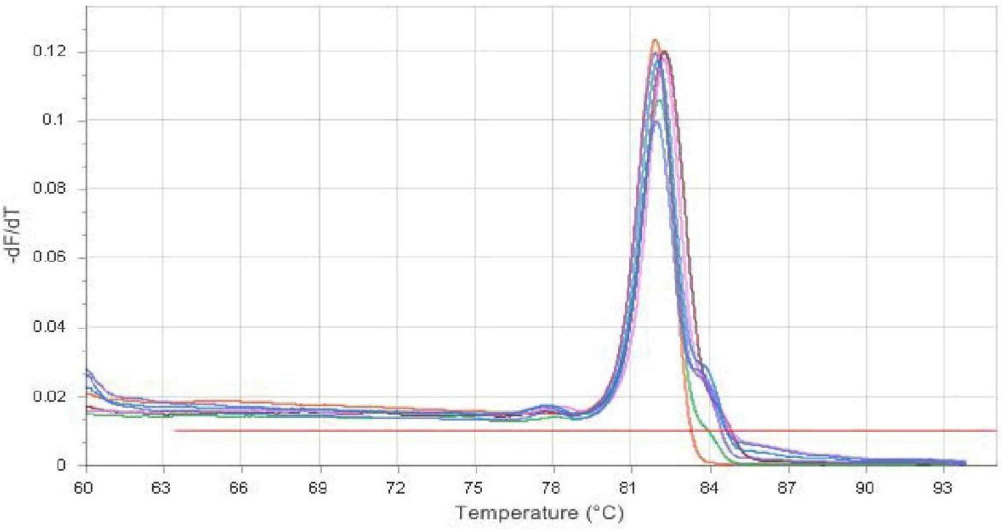

Melting curve corresponding *Hexb* gene.

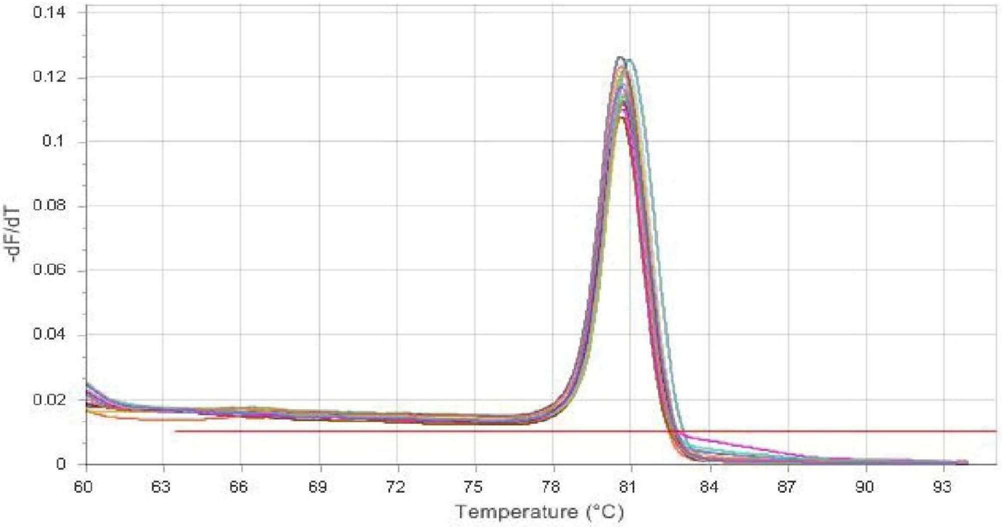

Melting curve corresponding *Slamf6* gene.

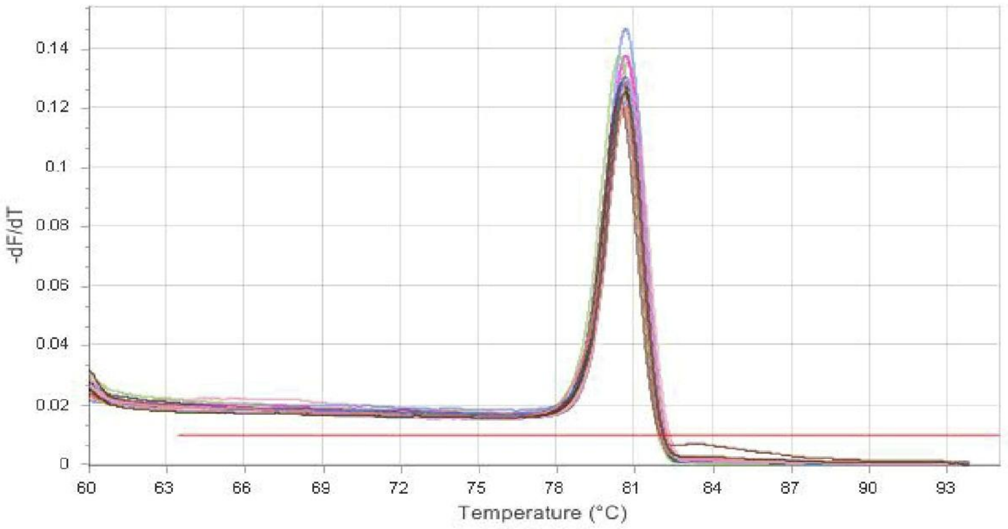

Melting curve corresponding *Slc14a1* gene.

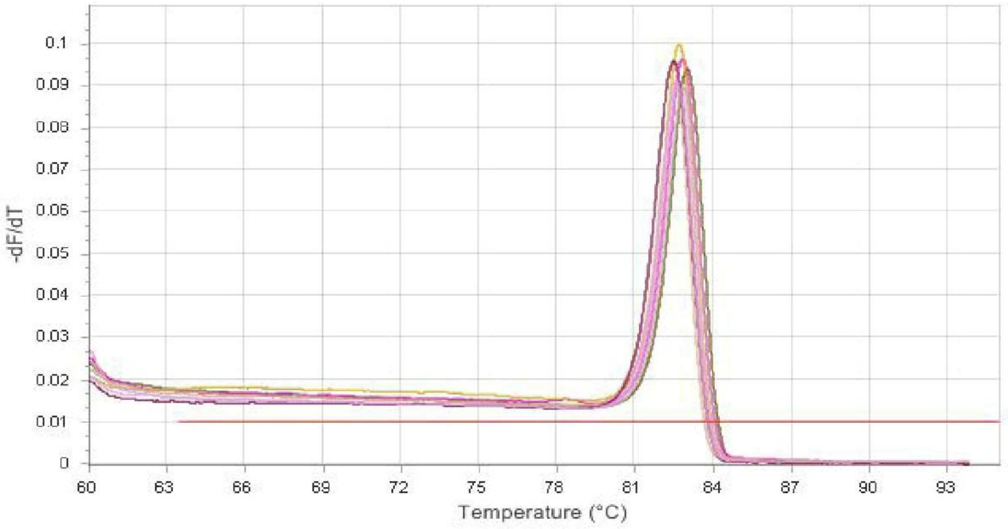

Melting curve corresponding *Rn18s* gene.

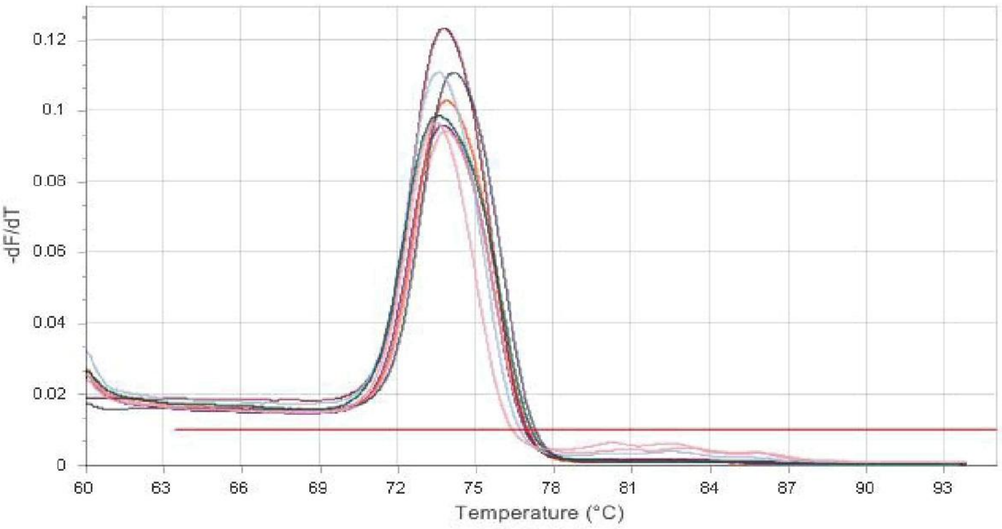

S2 File-Section B: Results of the reverse PCR. Only one product with a size equivalent to the corresponding amplicon could be amplified by each primer.

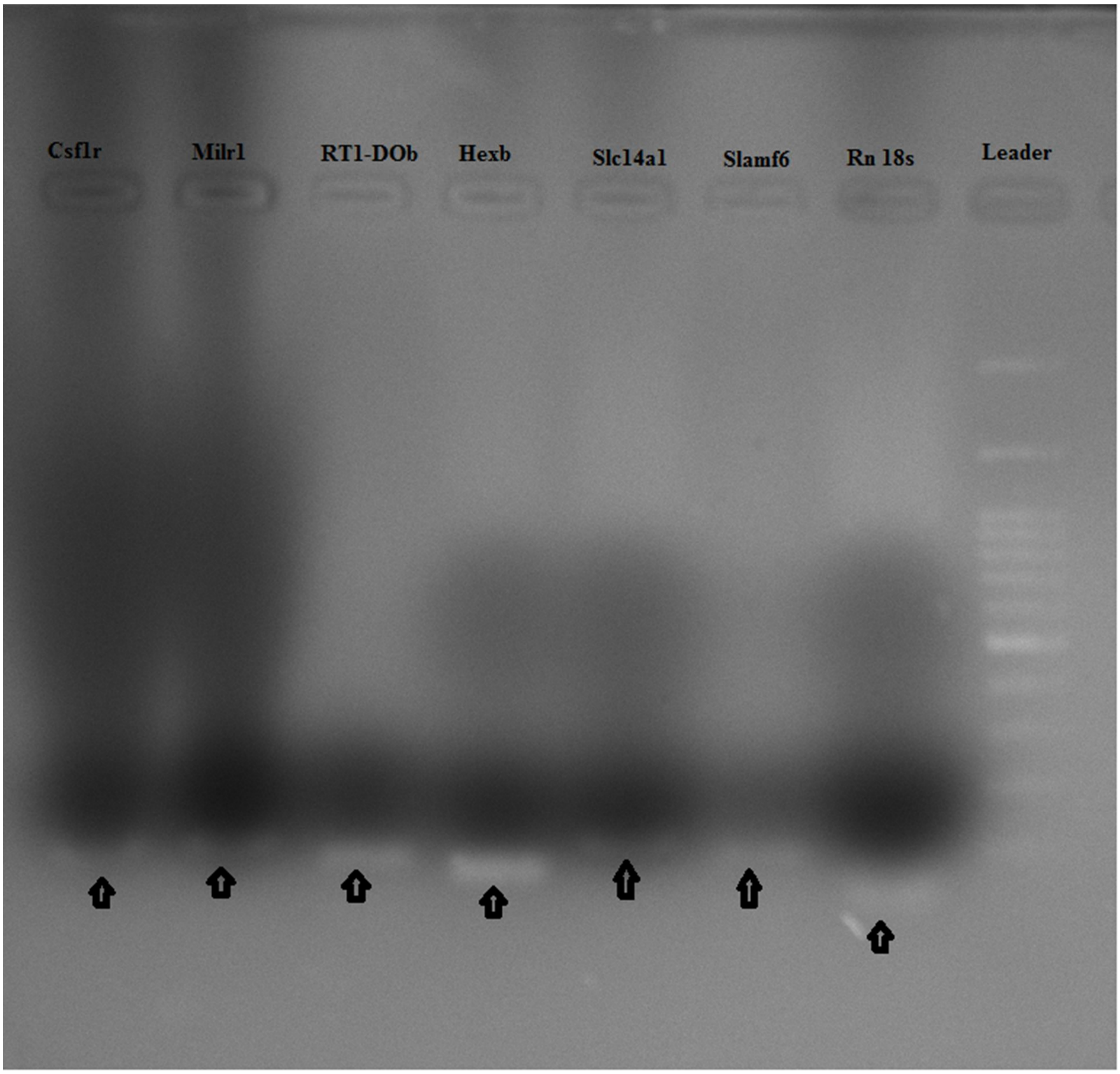

S3 File: The mean value of Ct for surveyed genes.

Sample	Cq (Csf1r)	Cq (Milr1)	Cq (RT1-DOb)	Cq (Rn 18s)	Cq (Hexb)	Cq (Slamf6)	Cq (Slc14a1)
A1	26.34166603	25.21393905	29.90153895	5.198675282	21.80783714	24.66342558	32.31711395
A2	34.26130681	31.84250196	28.17279065	4.172334099	20.86750644	24.50624351	30.67435571
A3	21.55653146	29.13205664	27.47989507	8.585304528	21.99077998	22.51256738	32.4208794
A4	22.99943622	31.94352437	30.73957793	5.051059214	23.41086433	24.8404927	33.17428495
A5	28.88337027	33.64168139	32.33530747	7.051032903	25.59657992	27.01802861	33.73395022
A6	22.90303044	26.20947996	27.72661181	10.44643811	20.9856032	29.84156849	33.49145053
A7	26.3871797	28.23956581	28.3985924	4.137663421	21.62170256	27.32148414	36.22253845
A8	23.86954865	29.35557052	27.87673034	4.431932371	19.18439606	25.35878239	31.22193931
A9	23.75174803	28.10423997	28.34608287	4.252420406	21.42882482	26.68224349	31.77529356
C1	23.33763764	26.3622304	28.39299475	16.34408749	23.40501619	24.72088966	31.19943848
C5	23.17436078	21.88420892	28.12388417	4.181772625	22.46483754	25.10426138	32.22965488
C2	23.51245491	29.22594097	28.15377445	9.48161472	22.04997871	23.49095745	34.77441467
C3	22.82788538	26.26068756	28.05312251	4.765930968	22.10855883	22.67518045	29.71709229
C4	28.93382372	32.37663149	32.39334683	8.481544456	25.0764513	28.94080799	36.05821187
C6	24.09365574	29.39776522	28.1662346	4.46235737	22.49482823	31.1720593	30.1383928
C7	22.58415425	25.57413609	26.45411904	4.008973467	22.34438479	27.24616282	31.04838588
C8	22.92058484	31.82166388	27.83094162	3.906119101	21.95007812	24.39418165	30.92938462
C9	22.47176988	29.60691687	28.22471146	4.86370855	19.1927105	25.76333632	32.0586472
